# Sirt6 opposes glycochenodeoxycholate-induced apoptosis of biliary epithelial cells through the AMPK/PGC-1α pathway

**DOI:** 10.1186/s13578-020-00402-6

**Published:** 2020-03-20

**Authors:** Jiye Li, Dongsheng Yu, Sanyang Chen, Yifan Liu, Jihua Shi, Jiakai Zhang, Peihao Wen, Zhihui Wang, Jie Li, Wenzhi Guo, Shuijun Zhang

**Affiliations:** 1 Department of Hepatobiliary and Pancreatic Surgery, The First Affiliated Hospital of Zhengzhou University, Zhengzhou, 450052 Henan China; 2 Henan Key Laboratory of Digestive Organ Transplantation, Zhengzhou, 450052 Henan China; 3 Zhengzhou Key Laboratory of Organ Transplantation Technology & Application Engineering, Zhengzhou, 450052 Henan China; 4 Academy of Medical Sciences, Zhengzhou University, Zhengzhou, 450052 Henan China; 5 Department of Traditional Chinese Medicine, The First Affiliated Hospital of Zhengzhou University, Zhengzhou, 450052 Henan China

**Keywords:** Sirt6, HiBEC, Cholestasis, PGC-1α, AMPK

## Abstract

**Background:**

Induction of biliary epithelial cell apoptosis by toxic bile acids is involved in the development of cholestatic disease, but the underlying molecular mechanism is not clear. The purpose of this study was to investigate the molecular mechanisms involved in Sirt6 protection against the apoptosis of human intrahepatic biliary epithelial cells (HiBEC) induced by the bile acid glycochenodeoxycholate (GCDC).

**Results:**

Sirt6 was either overexpressed or knocked down in HiBEC, with or without GCDC pretreatment. The CCK-8 assay was used to assess cell viability and, Hoechst 33258 staining was used to determine apoptotic rate. Mitochondrial DNA (mtDNA) copy number, malondialdehyde (MDA) and reactive oxygen species (ROS) production were detected to evaluate the severity of the mitochondrial dysfunction and oxidative stress. The mRNA and protein levels of PGC-1α, Nrf1, and Nrf2 were analyzed using RT-qPCR and western blot assay. The results showed that Sirt6 opposed GCDC-induced apoptosis in HiBEC via up-regulating PGC-1α expression and stabilizing mtDNA. We used agonists and inhibitors of AMPK to demonstrate that Sirt6 increased PGC-1α expression through the AMPK pathway whereas GCDC had the opposite effect. Finally, western blot, luciferase assay, and co-immunoprecipitation were used to describe a direct interaction and acetylation modification of PGC-1α by Sirt6.

**Conclusion:**

Our data illuminated that Sirt6 ameliorated GCDC-induced HiBEC apoptosis by upregulating PGC-1α expression through the AMPK pathway and its deacetylation effect.

## Introduction

In cholestatic liver disease, both biliary epithelial cells and hepatocytes are exposed in their microenvironment to toxic bile acids arising from the disruption of bile acid homeostasis [1]. Hydrophobic bile acids such as glycochenodeoxycholate (GCDC) are highly cytotoxic and can induce both hepatocyte and bile duct epithelial cell apoptosis. However, the mechanism of bile acid-induced bile duct epithelial cells injury needs to be elucidated both to alleviate biliary and liver injury and to assist in the discovery of new biomarkers.

Sirt6, a member of the mammalian sirtuin family, is a NAD^+^-dependent deacetylase that modifies the 9, 18 and 56 lysine residues of histone H3 (H3K9ac, H3K56ac and H3K18ac) [2, 3] and the lysine residues of many other transcription factors [4–6]. These functions make Sirt6 an important epigenetic regulator of chromatin and nuclear signaling systems, involved in heterochromatin silencing and genome maintenance, differentiation regulation, tumor suppression, and regulation of glycolipid metabolism balance [7–10].

Mitochondria are energetic organelles that rely on respiratory chain electron transport to produce ATP through oxidative phosphorylation. When mitochondrial dysfunction occurs, electrons overflow from the respiratory chain and react with oxygen in a non-physiological pattern to produce reactive oxygen species (ROS) and promote the induction of apoptosis. In cholestatic disease, mitochondrial dysfunction and oxidative stress interact with each other and are important factors that induce cell death [11]. Growing evidence indicates that bile acid impairs hepatocyte mitochondrial function and contributes to cholestasis [12]. Thus, preventing oxidative stress and mitochondrial dysfunction in hepatocyte and biliary epithelial cells can improve hepatic function and reduce cholestasis [13]. Bile acids inhibit mitochondrial respiratory chain complex activity and increase hepatocyte production of reactive oxygen species. Transcription factors involved in mitochondrial biosynthesis include mitochondrial transcription factor A (TFAM), nuclear respiratory factor (NRF-1/NRF-2) and peroxidase-activated receptor gamma activator-1α (PGC-1α). PGC-1α is the major regulator of mitochondrial biogenesis and function, and can enhance the expression of NRF1 and NRF2 and the transcriptional activity of TFAM [14].

Previous studies showed that Sirt1 is a transcriptional and transactivational regulator of murine farnesoid X receptor (FXR), the primary bile acid sensor, and as such could reverse the cholic acid feeding-induced liver injury in mice [15]. Furthermore, Sirt7 promoted the proliferation and cell cycle progression of cholangiocarcinoma cell lines in vitro and in vivo by induce the p21 expression and increased the levels of Cyclin D1 and cyclin dependent kinase 2 [16]. Sirt6 also has been revealed to play important roles in protecting against ischemia/reperfusion injury in multiple organs including liver [17].

Despite the ability of Sirt6 to modulate liver injury and metabolism, very few studies have focused on the effect of Sirt6 on biliary epithelial cells in cholestasis disease. The current study aims to: (1) confirm the decrease in expression of Sirt6 in cholestatic biliary epithelial cell injury, (2) determine whether activation of Sirt6 alleviates cholestatic biliary epithelial cell injury and, if so, (3) whether this is achieved by regulating the expression of PGC-1α and, if so, through what mechanism.

## Material and methods

### Cell culture

Human intrahepatic biliary epithelial cells (HiBEC) were purchased from ScienCell (San Diego, CA) and maintained at 37 °C in 5% CO_2_ in RPMI 1640 supplemented with 10% fetal bovine serum (FBS), 100 U/mL penicillin, 100 mg/mL streptomycin and 2 mM glutamine. For Sirt6 overexpression, HiBEC cells were cultured in 6-well plates transfected with pcDNA3-HA or Sirt6 using lipo2000 for 48 h. For Sirt6 knockdown assay, cells were transfected with 100 nM Sirt6 or nonspecific siRNA in RPMI 1640 for 48 h. AICAR (#S1802), Compound C (#S7840) and L-NAME (#S2877) were obtained from Selleck (Shanghai, China).

### Cell viability

CCK-8 assay was used to determine the effect of treatment on cell viability. HiBEC were inoculated into 96-well plates at 5 × 10^4^ cells/mL, treated with GCDC (#G0759, Sigma-Aldrich) at the indicated concentrations and times, and 5 μL CCK-8 (Med Chem Express, Shanghai, China) was added into the cell culture medium. After further 2 h incubation, the optical density (OD) at 450 nm was measured using a microplate reader (Varioskan LUX, Thermo Fisher, USA).

### ROS and malondialdehyde (MDA) assays

For assay of ROS, the superoxide indicator dihydroethidium (DHE, Invitrogen) was added at 5 μM into HiBEC culture medium and incubate for 1 h at 37 °C. Fluorescence was measured according to kit instructions with 485 nm excitation and 527 nm emission.

Levels of MDA, a product of lipid peroxidation, were measured based on reaction between MDA and thiobarbituric acid with commercial kit from Beyotime Biotechnology (Shanghai, China) [18]. The enzymatic activities were recorded as units per milligram of protein (U/mg protein). Values obtained were the average of three independent measurements.

### Immunoprecipitation (IP) and immunoblotting

HiBEC cells were transfected with Flag-tagged Sirt6 and HA-tagged PGC-1α using lipo2000. After 48 h, total lysates were obtained by lysing in ice-cold lysis buffer (50 mM Tris–HCl, pH 7.5, 0.5% Nonidet P-40, 150 mM NaCl, 2 mM EGTA, 1 mM Na_3_VO_4_, 100 mM NaF, 10 mM Na_4_P_2_O_7_, 1 mM phenylmethylsulfonyl fluoride, 10 mg/mL aprotinin, 10 mg/mL leupeptin) [19]. Protein concentration was measured by the BCA assay (Bio-Rad, Hercules, CA, USA). IP of endogenous PGC-1α or Sirt6 were incubated with primary antibody of Sirt6 (#12486, CST) or PGC-1α (ab54481, Abcam) at 4 °C for 2 h respectively and with protein A+G sepharose beads (P001-3, 7-Sea Biotech, Shanghai, China) for an additional hour at 4 °C. IP of Flag-tagged Sirt6 or HA-tagged PGC-1α were incubated with Anti-Flag M2 affinity Gel (#F2426, Sigma-Aldrich) or anti-HA agarose (#26181, Thermo Fisher) for 3 h respectively. The immunocomplexes were washed three times with lysis buffer and boiled at 100 °C for 5 min in loading buffer (#P0015B, Beyotime Biotechnology, Shanghai, China) and separated in 10% SDS-PAGE gels and transferred to nitrocellulose membranes (#IPFL00010, Millipore, Bedford, MA, USA). The membranes were blocked with 5% non-fat milk and incubated with specific antibodies overnight at 4 °C. β-actin and COX IV were used as the cytoplasm and mitochondrial loading control respectively. Primary antibodies were used at the dilution of 1:1000. Antibodies to Phospho-Akt (Ser473) (#4060), Akt (#4685), ERK1/2 (#4695), Phospho-ERK1/2 (#4370), COX IV (#4850), Nrf1 (#46743), Nrf2 (#12721), cytochrome *c* (#11940), Flag M2 (#14793), HA(#3724), GAPDH(#2118) were purchased from Cell Signaling Technology (Beverly, MA, USA). Anti-β-actin (ab8266) and horseradish peroxidase-conjugated anti-mouse or rabbit IgG were purchased from Abcam (Cambridge, MA, USA).

### RT-qPCR

Total RNA was extracted from cells using RNAiso Plus (Takara Bio Inc., Dalian, China) and was reverse-transcribed using HiScript III RT SuperMix (R323-01, Vazyme, Nanjing, China) according to the manufacturer’s protocols. qPCR was performed using SYBR (#B21203, Bimake, Shanghai, China). Primer sequences used in the experiments were as follows:

**Table:** 

Gene	Forward (5′-3′)	Reverse (5′-3′)
homo-PGC-1α	TCTGAGTCTGTATGGAGTGACAT	CCAAGTCGTTCACATCTAGTTCA
homo-Nrf1	AGGAACACGGAGTGACCCAA	TATGCTCGGTGTAAGTAGCCA
homo-Nrf2	TCAGCGACGGAAAGAGTATGA	CCACTGGTTTCTGACTGGATGT
homo-TFAM	GACAGAGGTGGCTCAACAGC	CCTTGCGGGGGAGGAATAAG
homo-ND1	CTAATCGCAATGGCATTCCTAA	TGGTAGATGTGGCGGGTTTT
homo-ND6	AAAGTTTACCACAACCACCACCC	ATTGAGGAGTATCCTGAGGCATG
homo-COX I	ACTAACAGACCGCAACCTCAACA	CCGAAGCCTGGTAGGATAAGAAT
homo-mtDNA	CAAACCTACGCCAAAATCCA	GAAATGAATGAGCCTACAGA
homo-GAPDH	AATGGGCAGCCGTTAGGAAA	GCGCCCAATACGACCAAATC

The expression levels of mRNA were normalized to GAPDH. The reactions were incubated at 95 °C for 10 min, followed by 40 cycles of 95 °C for 15 s and 60 °C for 40 s.

### Vectors used

pGL3-PGC-1α containing PGC-1α ‒ 1000 to + 1 promoter was purchased from Genewiz (Suzhou, China), pCIP-AMPKα1-WT and pCIP-AMPKα1 (DN) were gifts from Reuben Shaw (Addgene plasmid # 79010 and #79011) [20]; pCMV4a-Sirt6-Flag was a gift from Hening Lin (Addgene plasmid # 102322) [21]; pcDNA4-myc-PGC-1α was a gift from Toren Finkel (Addgene plasmid # 10,974) [22]; and Sirt6 si-RNA (si-Sirt6, #116148) and the nonspecific control (si-NC) were purchased from Thermo Fisher.

### Luciferase assays

HiBEC cells were seeded onto 24-well plates, grown to 40–50% confluency and transfected with luciferase reporter plasmids using lipo2000. Briefly, cells were incubated for 4 h in 250 μL of serum-free RPMI-1640 containing pGL3-PGC-1α (200 ng), pRL-TK-Renilla (10 ng), AMPKα1 or AMPKα1(DN) (40 ng), and then grown in RPMI-1640 with 10% FBS for 48 h. Before harvest, the cells were incubated for 12 h in the complete medium described above supplemented with the AMP analog AICAR (1 mM) or the AMPK inhibitor Compound C (5 μM). Luciferase activity was measured using the Dual-Luciferase Reporter Assay System (#E1910, Promega, Madison, WI) and normalized to *Renilla* luciferase values. Measurements for three biological replicates were taken in triplicate and averaged.

### Statistical methods

The data were expressed as mean ± SEM. All experiments were performed in triplicate. All statistical analyses were performed with SPSS 19.0 using non-parametric tests. The Kruskall-Wallis test followed by the Mann–Whitney U test was used to detect differences between groups. *P* < 0.05 was considered statistically significant.

## Results

### GCDC induced the down-regulation of Sirt6 expression in HiBEC

**Fig. 1:**
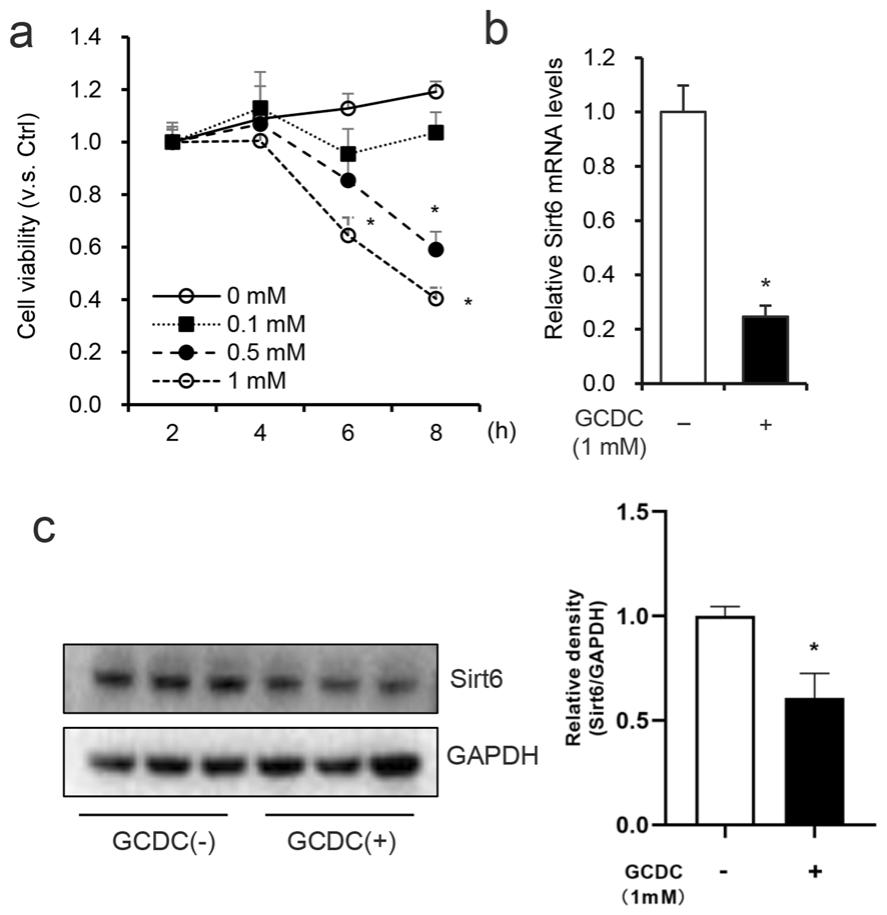
GCDC treatment induced the down-regulation of Sirt6. **a** HiBEC were treated with or without GCDC at the indicated concentrations and times, cell viability was determined using CCK-8 assay. **b**, **c** HiBEC were treated with 1 mM GCDC for 8 h, Sirt6 mRNA and protein levels were detected with RT-qPCR and western blot assay. *P < 0.05

HiBEC were treated with GCDC at the concentrations and times indicated in Fig. 1a and CCK-8 assay was used to assess cell viability. As shown in Fig. 1a, 1 mM GCDC treatment for 8 h significantly decreased HiBEC cell viability; this treatment condition was used in the following experiments. To unravel the biological significance of Sirt6 on GCDC-induced HiBEC injury, we next analyzed the Sirt6 mRNA and protein expression levels in HiBEC and found that 1 mM GCDC significantly decreased Sirt6 mRNA and protein expression (Fig. 1b, c). This was consistent with previous research results that bile duct ligated models of cholestasis demonstrate decreased another sirtuin family member Sirt1 protein expression in liver [23].

### Sirt6 ameliorated the GCDC-induced HiBEC apoptosis

**Fig. 2:**
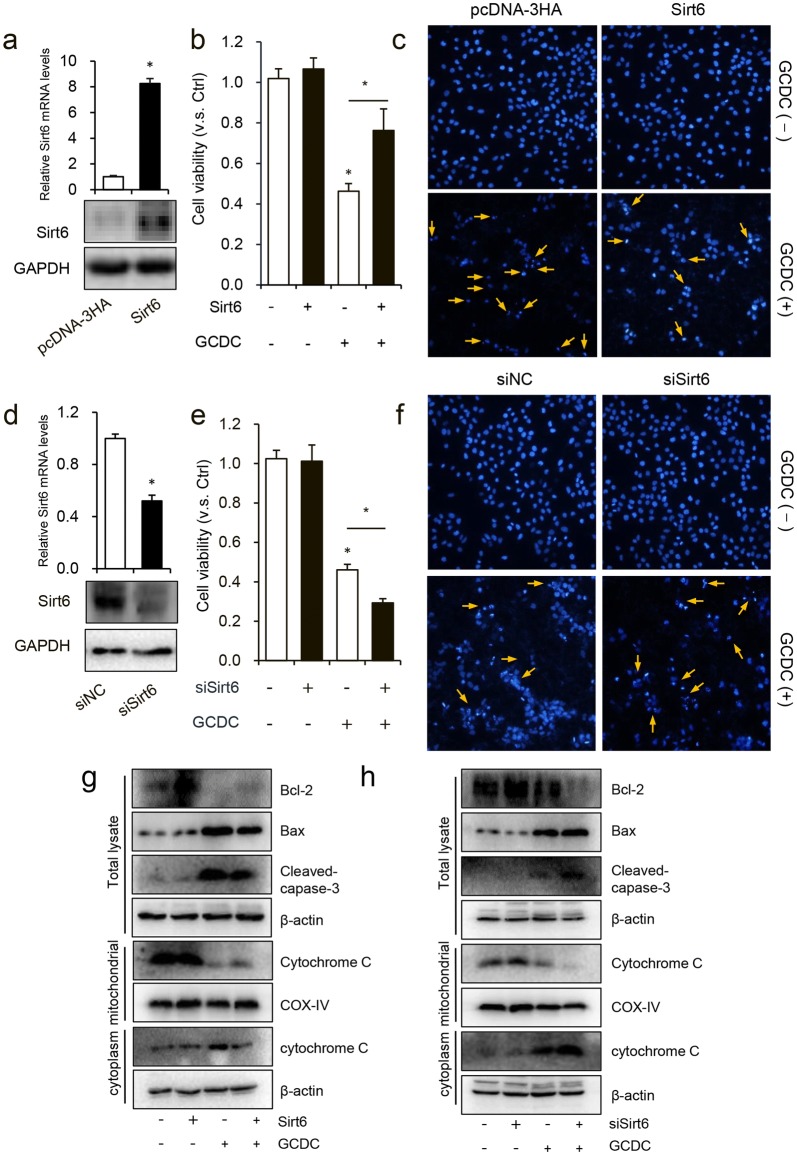
Sirt6 ameliorated GCDC-induced HiBEC apoptosis. **a**–**c** HiBEC were transfected with pcDNA3-HA or Sirt6 expression vector for 48 h, **a** Sirt6 mRNA and protein levels were analyzed using RT-qPCR and western blot assay. Before harvesting cells were treated with or without 1 mM GCDC for 8 h, **b** the CCK-8 assay was used to assess cell viability and, **c** Hoechst 33,258 staining was used to determine apoptotic rate. **d**–**f** HiBEC were transfected with si-NC or si-Sirt6 for 48 h, **d** Sirt6 mRNA and protein levels were analyzed using RT-qPCR and western blot assay. Before harvesting cells were treated with or without 1 mM GCDC for 8 h, the cell viability and apoptotic rate were detected with **e** CCK-8 assay and **f** Hoechst33258 staining, respectively. **g**, **h** total cell lysates, mitochondrial and cytoplasm lysates were harvested for western blot assay with indicated antibodies. *P < 0.05

Next, we sought to identify the effects of Sirt6 on GCDC-induced cell injury on HiBEC. HiBEC were transfected with vector control or Sirt6 expression vector for 48 h; then, before harvest, cells were treated with 1 mM GCDC for 8 h. Sirt6 transfection significantly elevated Sirt6 mRNA and protein levels (Fig. 2a). Cell viability and apoptosis were analyzed with CCK-8 assay and Hoechst-33258 staining and revealing that Sirt6 overexpression increased cell viability when compared with vector control (Fig. 2b) and decreased apoptosis (Fig. 2c).

Knockdown of Sirt6 was carried out with transfection of si-Sirt6 into HiBEC. The transfection effect was detected with RT-qPCR and western blot assay (Fig. 2d). Sirt6 knockdown significantly decreased cell viability and increased the apoptotic rate (Fig. 2e, f).

Next, we examined whether Sirt6 protected against GCDC injury by blocking apoptotic cell death. We detected the expression levels of cleaved caspase-3, Bax and Bcl-2, which are regarded as markers of apoptosis, by western blot analysis and found that GCDC treatment caused significant increase of the apoptosis markers compared with control group. Sirt6 overexpression significantly decreased the levels of cleaved caspase-3 and Bax and increased the expression of Bcl-2, while knockdown of Sirt6 had the opposite effect, confirming the anti-apoptotic activity of Sirt6 after GCDC injury.

During apoptosis, cytochrome *c* release from mitochondria is dependent on either the Bcl-2 family members Bax and Bak and/or on the mitochondrial permeability transition pore (MPTP) [24]. The mitochondrial and cytoplasmic proteins were isolated for western blot assay, and showing that Sirt6 overexpression decreased and knockdown increased cytochrome *c* release from mitochondria into the cytoplasm (Fig. 2g, h).

### Sirt6 opposed the GCDC-induced mtDNA injury

**Fig. 3:**
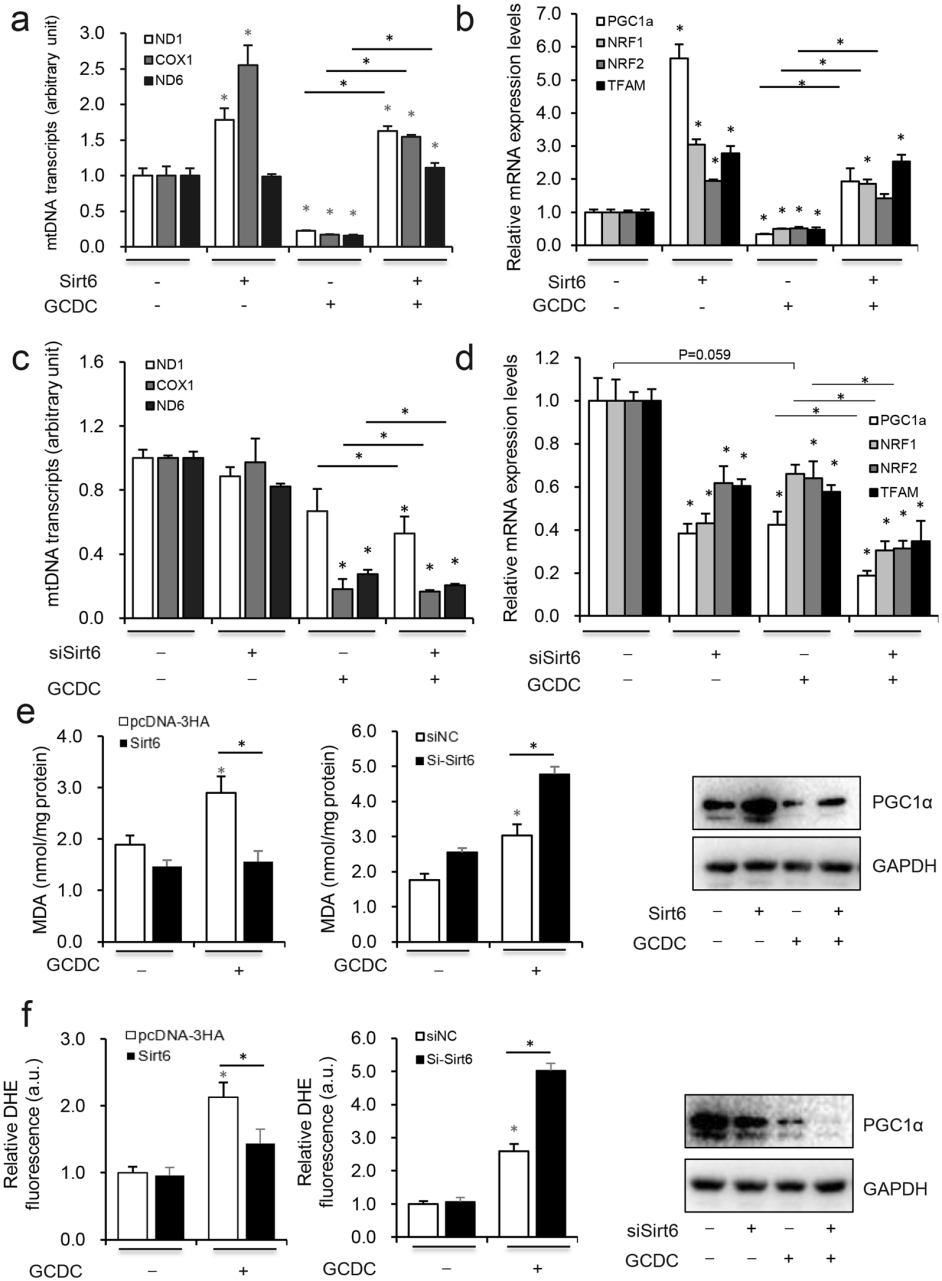
Sirt6 opposed the GCDC-induced mtDNA injury and induce the PGC-1α expression. HiBEC were transfected with **a**, **b** pcDNA3-HA or Sirt6, **c**, **d** si-NC or si-Sirt6 for 48 h, before harvesting cell were treated with 1 mM GCDC for 8 h, *ND1*, *COX1*, *ND6*, *TFAM*, *PGC-1α*, *Nrf1*, *Nrf2* mRNA levels were analyzed using RT-qPCR. **e**–**f** MDA, ROS were assayed and the PGC-1α protein levels were detected with western blot assay. *P < 0.05

Oxidative stress and mitochondrial dysfunction are involved in the pathogenesis of cholestasis. Mitochondrial DNA is highly sensitive to oxidative stress and susceptible to oxidative damage. Once mtDNA damage exceeds a certain threshold, mtDNA amplifies oxidative stress through its encoded respiratory chain subunit, which aggravates mitochondrial oxidative damage until cell death. Here, the genes ND1, ND6 and COX1 were used to determine the mtDNA copy number [25] and we found that GCDC treatment significantly decreased the mtDNA copy number, Sirt6 overexpression reversed and knockdown increased the negative effect of GCDC (Fig. 3a, c). PGC-1α and NRF1, NRF2, and TFAM are known as mtDNA proliferation markers [14] and their mRNA and protein levels were detected with RT-qPCR and western blots. GCDC treatment significantly decreased whereas Sirt6 overexpression increased these markers and knockdown increased the effect (Fig. 3b, d). GCDC treatment significantly increased levels of cell ROS and MDA, consistent with previous reports [26, 27], while Sirt6 inhibited and Sirt6 knockdown increased GCDC-induced oxidative stress (Fig. 3e, f).

### Sirt6 induced PGC-1α activation through AMPK pathway

**Fig. 4:**
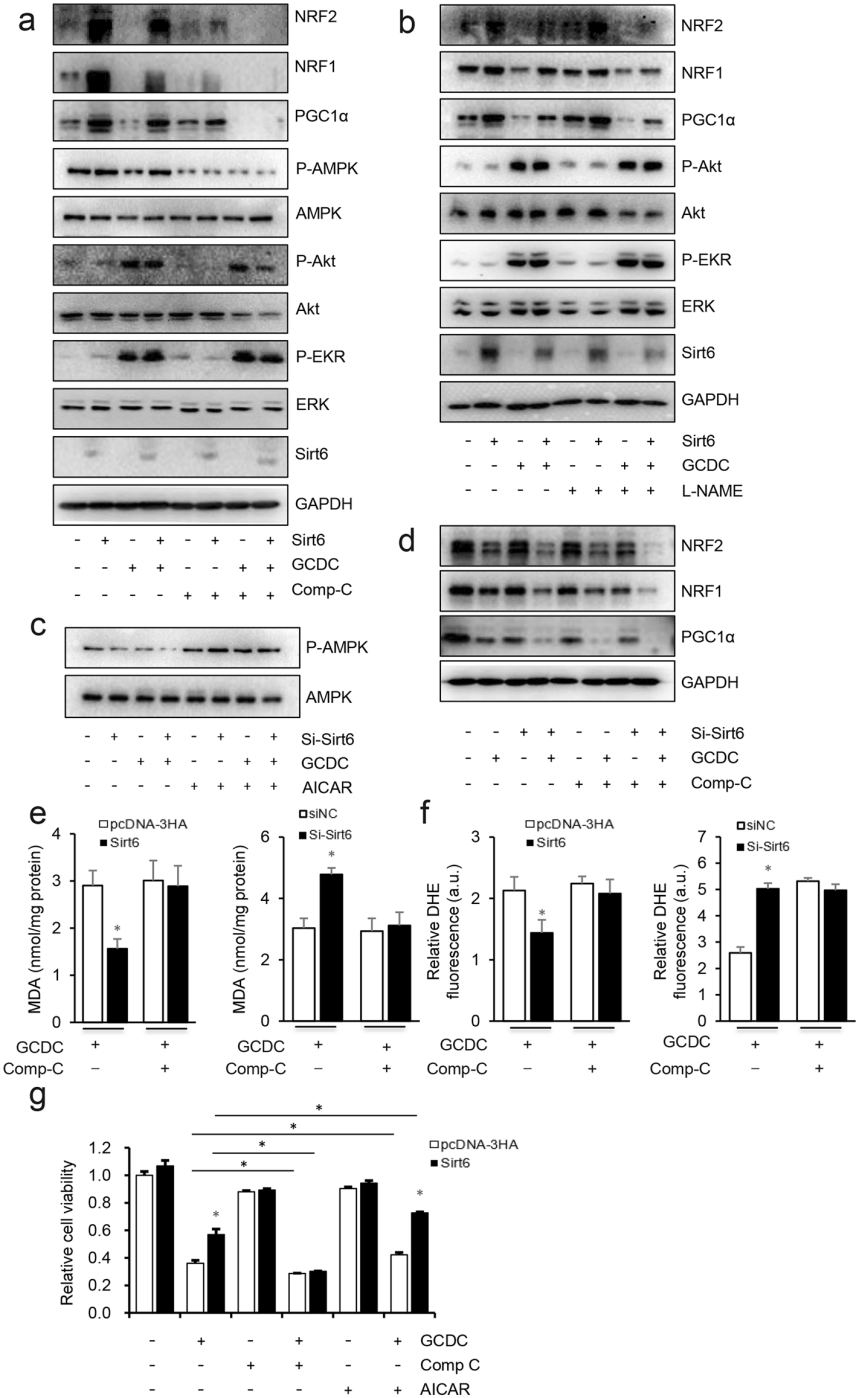
Sirt6 induced the expression of PGC-1α through AMPK pathway. **a**, **b** HiBEC were transfected with pcDNA3-HA or Sirt6 for 48 h, before harvest, **a** 5 μM Comp C or **b** 500 μM L-NAME were preincubated for 12 h prior to stimulation with GCDC (1 mM) for 8 h, total cell lysates were immunoblotted with indicated antibodies. **c**, **d** HiBEC were transfected with si-NC or si-Sirt6 for 48 h, before harvest, **c** 1 mM AICAR or **d** 5 μM Comp C were preincubated for 12 h prior to stimulation with GCDC (1 mM) for 8 h, total cell lysates were immunoblotted with indicated antibodies. **e** MDA and **f** ROS were assayed. **g** HiBEC were transfected with pcDNA3-HA or Sirt6 for 48 h, before harvest, cells were treated with 1 mM AICAR or 5 μM Comp C for 12 h, then CCK-8 assay was used to detect the cell viability. *P < 0.05

Based on the role of Sirt6 in improving mitochondrial function, we hypothesized that Sirt6 may be involved in the regulation of mitochondrial biogenesis. Our results also demonstrated that Sirt6 upregulated the mitochondrial biosynthesis regulator PGC-1α, which in turn enhanced the expression of downstream NRF1, NRF2 and TFAM. As previously reported, the expression of PGC-1α is regulated by several agents including nitric oxide synthase (NOS), adenosine 5′-monophosphate (AMP)-activated protein kinase (AMPK), Akt and EKR1/2, which are also regulated by Sirt6. Accordingly, we used the AMPK inhibitor Comp C and the NOS inhibitor L-NAME to determine their effects of Sirt6-induced up-regulation of PGC-1α. HiBEC were transfected with Sirt6 and then treated with GCDC, Comp C or L-NAME. We found that GCDC treatment decreased the phosphorylation of AMPK and the expression of PGC-1α, Nrf1 and Nrf2, whereas Sirt6 significantly increased the phosphorylation of AMPK and the expression of PGC-1α signaling pathway members. Treatment with Comp C completely prevented the effects of Sirt6 on AMPK and PGC-1α, whereas L-NAME had no effect (Fig. 4a, b). Activation of PI3K/AKT signaling and ERK1/2 is known to be required for PGC-1α expression and activity [28, 29]. We found that GCDC significantly increased Akt and Erk1/2 phosphorylation, and that Sirt6 had no effect. We verified these results in Sirt6 knockdown HiBEC cells (Fig. 4c, d). In addition, the effects of Sirt6 on GCDC-induced ROS accumulation, MDA up-regulation were all prevented by the AMPK inhibitor Comp C (Fig. 4e, f), indicating that Sirt6 maintained mtDNA integrity and mitochondrial function and inhibited oxidation through the AMPK pathway. We further tested the effects of the AMPK agonist AICAR and inhibitor Comp C along with Sirt6 or GCDC on cell viability to test whether AMPK affected cell viability. As expected, Comp C treatment significantly decreased and AICAR increased the cell viability, and transfection with Sirt6 collaborated with AICAR to increase the cell viability (Fig. 4g). From these results, we concluded that Sirt6 regulated the expression of PGC-1α and protects against GCDC-induced injury through AMPK pathway.

### Sirt6 directly interacted with and deacetylates PGC-1α

**Fig. 5:**
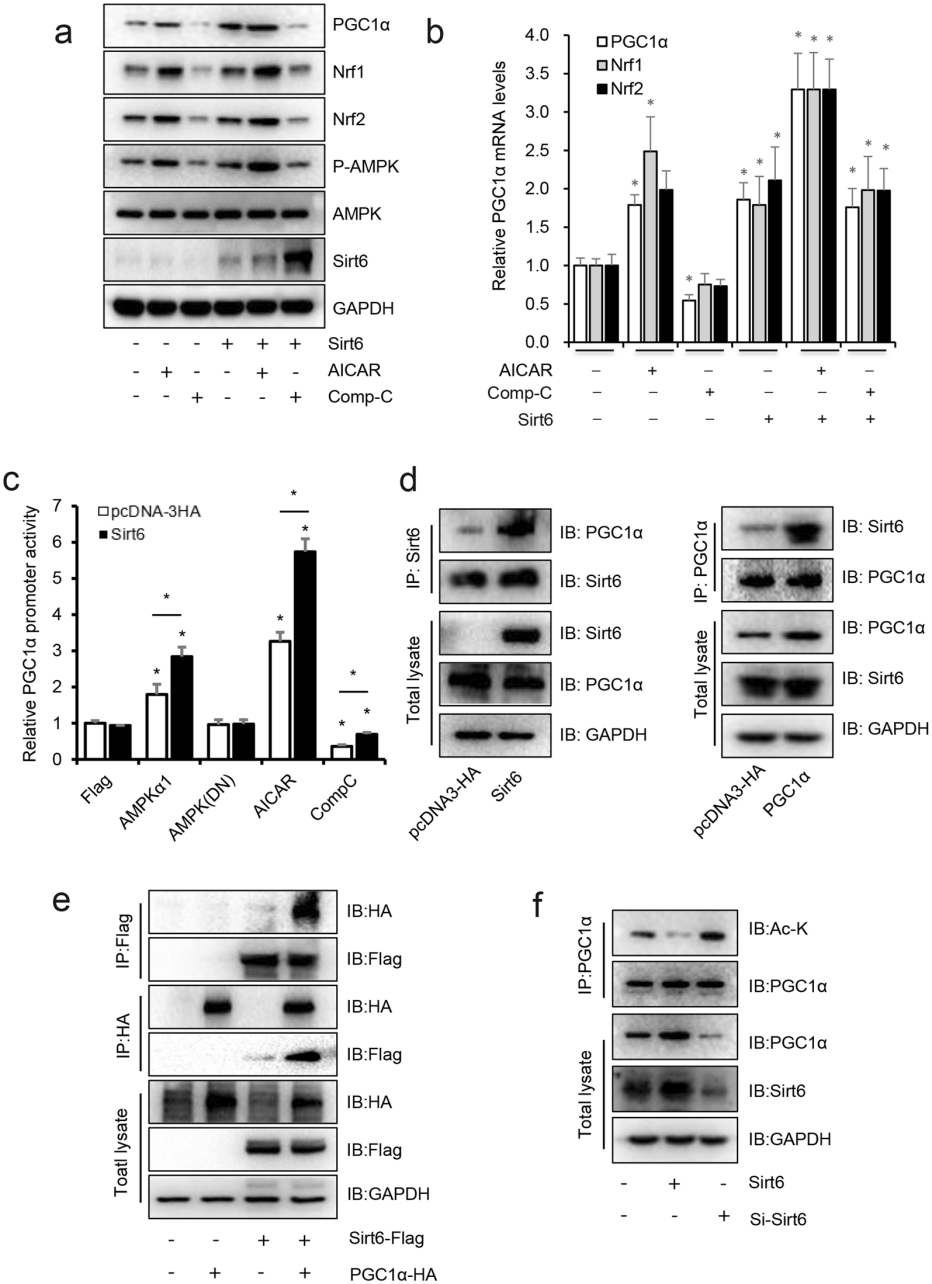
Sirt6 directly interacted with and deacetylates PGC-1α. **a**, **b** HiBEC were transfected with pcDNA3-HA or Sirt6 for 48 h, before harvest, then treated with 1 mM AICAR or 5 μM Comp C for 12 h, cells were harvested for western blot and RT-qPCR assay. **c** HiBEC were transfected with ‒ 1000/ + 1-LUC PGC-1α construct (200 ng) plus pRL-TK-Renilla (10 ng), pcDNA3-HA (200 ng), Sirt6 (200 ng), AMPKα1 (200 ng) and AMPKα1 (DN) (200 ng), treated with or without 1 mM AICAR or 5 μM Comp C for 12 h. PGC-1α promoter driven luciferase activity were normalized to Renilla luciferase activity, presented as mean ± SEM. **d** HiBEC were transfected with vector control, Sirt6 or PGC-1α for 48 h, total lysates were harvest, immunoprecipitated with antibody to Sirt6 or PGC-1α, and immunoblotted with antibodies to PGC-1α and Sirt6. **e** Flag-Sirt6 was co-expressed with HA-tagged PGC-1α, immunoprecipitated with antibody to Flag or HA, and immunoblotted with antibodies to HA or Flag. Cell extracts were also immunoblotted with antibodies to HA, Flag or GAPDH. **f** HiBEC cells were transfected with Sirt6 or si-Sirt6 for 48 h, total cell lysates were immunoprecipitated with antibody to PGC-1α then immunoblotted with antibody to Ac-K. *P < 0.05

To further evaluate the role of AMPK in the Sirt6 regulated PGC-1α expression, we overexpressed Sirt6 and treated with AICAR or Comp C in HiBEC. Western blot and RT-qPCR were used to detect the PGC-1α protein and mRNA levels. As shown in Fig. 5a, b, overexpression of Sirt6 and activation of AMPK with AICAR significantly induced the PGC-1α, NRF1 and NRF2 protein and mRNA levels, while Comp C significantly inhibited the effect of Sirt6.

As expect, our reporter gene assay performed in HiBEC demonstrated that Sirt6 treatment significantly increased the PGC-1α promoter-driven luciferase, while co-treatment with Comp C decreased it, and that addition of AICAR or overexpress AMPK increased PGC-1α transcription activity. Meanwhile, the dominant-negative α1-subunit of AMPK (AMPK(DN)) was unable to induce the activation of PGC-1α (Fig. 5c).

After identifying PGC-1α as a potential target for Sirt6, we suspected that, as a histone deacetylase, Sirt6 might directly bind to its target gene and affect protein complex formation through epigenetic modification. Using reciprocal immunoprecipitation, we confirmed the interaction between Sirt6 and PGC-1α in both endogenous and exogenous expression levels (Fig. 5d, e). We also found that overexpression of Sirt6 significantly decreased and knockdown increased the acetylation of PGC-1α (Fig. 5f), consistent with previous reports [30].

## Discussion

Cholestatic liver disease such as primary biliary cirrhosis, primary sclerosing cholangitis, and graft-versus-host disease often cause biliary epithelial cell damage [31]. Toxic bile acids can induce apoptosis and necrosis of biliary epithelial cells. Most research is focused on the damage of hepatocytes, but little is focused on the biliary epithelial cells that also play an important role in the occurrence and development of the cholestasis. Biliary epithelial cells have multiple functions in the hepatobiliary system, participating in bile acid secretion and reabsorption, drug metabolism and promoting liver regeneration [32]. Thus, investigating the mechanism of bile acid-induced apoptosis of biliary epithelial cells will provide new strategies and targets for the treatment of cholestasis.

Sirt6 is widely associated with in a variety of disease including ischemia/reperfusion and alcoholic hepatic injury [17, 33], cardiovascular disease [34], kidney disease [35] and hepatocellular carcinoma [36]. Given the important role of the Sirtuin family in regulating biliary epithelial cell proliferation and bile acid metabolism [14, 15], we hypothesized that Sirt6 might play an important role in regulating bile acid-induced apoptosis of biliary epithelial cells.

Here, we determined that the level of Sirt6 was obviously decreased in GCDC-treated HiBEC. Our data also revealed that overexpression of Sirt6 could prevent the GCDC-induced apoptosis by stabilizing mtDNA and decreasing the production of ROS. GCDC treatment significantly inhibited the mRNA and protein expression levels of PGC1-α, while Sirt6 induced PGC-1α expression.

Bile duct ligation and bile acid treatment cause oxidative stress and the production of ROS [37], then cause damage to mitochondrial DNA. Previously studies found that PGC-1α, a 91 kDa transcription factor, played a central role in the regulation of metabolism. PGC-1α expression is increased in response to nutritional deficiency, hypoxia and cyclic adenosine monophosphate (cAMP) activation [38], which regulates mitochondrial DNA replication and cellular oxidative metabolism [39]. It has been reported that PGC-1α played a key role in mitochondrial biogenesis and its expression level was rate limiting for mitochondrial gene expression. PGC-1α cooperates with NRFs, including NRF1 and NRF2, and promotes the expression of TFAM to regulate mitochondrial biogenesis [40]. So, we hypothesized that Sirt6 protected against GCDC-induced HiBEC injury by upregulating the PGC-1α expression.

The expression of PGC-1α is regulated by a series of proteins (NOS, P38, ERK1/2, Akt, CaN, CaMK, CDK, AMPK) [41, 42], among which nitric oxide synthase (NOS) [43], adenylate-activated protein kinase (AMPK) [44], Akt [45] and ERK1/2 [46] are regulated by Sirt6. Our results showed that the NOS inhibitor L-NAME did not affect the expression of PGC-1α induced by Sirt6, but the AMPK inhibitor Comp C not only blocked the effect of Sirt6 on PGC-1α but also promoted the mtDNA damage, ROS accumulation, and MDA upregulation induced by GCDC. We verified the above conclusions by knockdown of Sirt6 or activation of AMPK using AICAR in HiBEC. In the absence of GCDC, overexpression of Sirt6 or AMPK activation also could induce the PGC-1α mRNA and protein expression by active its transcription activity.

Previously studies have showed that, Sirt1 physically interacted with and deacetylated PGC-1α to promote mitochondrial biogenesis in muscle cells [30, 47]. Surprisingly, Sirt6 uniquely induced an increase in the acetylation of PGC-1α through the direct modification [42]. However, the interaction between Sirt6 and PGC-1α in biliary epithelial cells has not yet been investigated. We therefore tested the direct interaction between Sirt6 and PGC-1α by mutual co-IP experiments both in endogenous and exogenous levels. In this assay, a Flag-tagged Sirt6 and HA-tagged PGC-1α were overexpressed in HiBEC and immunoprecipitated using the Flag and HA antibodies respectively. Flag-Sirt6 was successfully pulled down, and PGC-1α was clearly detectable in the IP sample, indicating a direct interaction between Sirt6 and PGC-1α in the HiBEC. In the further in vitro deacetylation assay, Sirt6 demonstrated strong deacetylase activity toward PGC-1α, and knockdown of Sirt6 significantly increased its acetylation. It is reported that Sirt6 induced PGC-1α acetylation though General Control Nonrepressed Protein 5 (GCN5) and suppressed glucose production in hepatic cells [7], which is in contrast to our study. We speculate to be due on one hand to the difference between hepatocytes and biliary epithelial cells, and on the other to the difference in the function of PGC-1α in regulating glucose metabolism and regulating mitochondrial biogenesis.

At present, the drugs for cholestatic liver disease include ursodeoxycholic acid (UDCA) and *S*-adenosyl-l-methionine (SAM). However, due to the complex pathogenesis, the current treatment effect is not satisfactory, and it is urgent to find new therapeutic targets. Some scholars have proposed the descending pathological model of cholestasis. According to this theory, early or primary lesions are usually located in the “downstream” bile duct, and the main pathogenesis may be immune-mediated bile duct necrotizing inflammatory injury. When cholestasis occurs, bile acids (especially hydrophobic bile acids) mediate the toxic effects, which will lead to the “upstream” bile duct and liver parenchyma damage [48]. Our research showed that Sirt6 could reduce bile acid-induced apoptosis of HiBEC. Therefore, Sirt6 might become a new gene target for the treatment of cholestatic liver disease. In recent years, researchers have synthesized and screened pyrrolo[1,2-a]quinoxaline derivatives, and obtained the first synthetic Sirt6 activator [49]. The further study of Sirt6 will bring new hope for the treatment of cholestasis.

## Conclusions

Our results demonstrated that GCDC treatment induced cell apoptosis, inhibited mitochondrial DNA stability and activated oxidative stress by decreasing PGC-1α expression. Sirt6 activated the PGC-1α/NRF1, NRF2 pathway by activating AMPK phosphorylation and directly deacetylating PGC-1α, which revealed Sirt6 as a promising therapeutic target in cholestasis-induced biliary epithelial cell injury.

## Data Availability

All the data is contained in the manuscript.

## Abbreviations

HiBEC: Human intrahepatic biliary epithelial cells
GCDC: Glycochenodeoxycholate
mtDNA: Mitochondrial DNA
MDA: Malondialdehyde
ROS: Reactive oxygen species
TFAM: Mitochondrial transcription factor A
NRF-1: Nuclear respiratory factor 1
NRF-2: Nuclear respiratory factor 2
PGC-1α: Peroxidase-activated receptor gamma activator-1α
FXR: Farnesoid X receptor
FBS: Fetal bovine serum
OD: Optical density
DHE: Dihydroethidium
IP: Immunoprecipitation
MPTP: Mitochondrial permeability transition pore
NOS: Nitric oxide synthase
AMPK: Adenosine 5′-monophosphate-activated protein kinase
AMPK(DN): Dominant-negative α1-subunit of AMPK
cAMP: Cyclic adenosine monophosphate
GCN5: General Control Nonrepressed Protein 5
UDCA: Ursodeoxycholic acid
SAM: *S*-Adenosyl-l-methionine
